# Sea foam as a source of fungal inoculum for the isolation of biologically active natural products

**DOI:** 10.1080/21501203.2014.931893

**Published:** 2014-07-09

**Authors:** David P. Overy, Fabrice Berrue, Hebelin Correa, Novriyandi Hanif, Kathryn Hay, Martin Lanteigne, Kathrine Mquilian, Stephanie Duffy, Patricia Boland, Ramesh Jagannathan, Gavin S. Carr, Marieke Vansteeland, Russell G. Kerr

**Affiliations:** ^a^ Nautilus Biosciences Canada Inc., Duffy Research Center, 550 University Ave., Charlottetown, PEI, CanadaC1A 4P3; ^b^ Department of Chemistry, University of Prince Edward Island, 550 University Ave., Charlottetown, PEI, CanadaC1A 4P3; ^c^ Department of Pathology and Microbiology, Atlantic Veterinary College, University of Prince Edward Island, 550 University Ave., Charlottetown, PEI, CanadaC1A 4P3; ^d^ Department of Biology, University of Prince Edward Island, 550 University Ave., Charlottetown, PEI, CanadaC1A 4P3; ^e^ Department of Biomedical Sciences, Atlantic Veterinary College, University of Prince Edward Island, 550 University Ave., Charlottetown, PEI, CanadaC1A 4P3

**Keywords:** sea foam, fungal diversity, antimicrobials, *Neosetophoma samarorum*, *Phaeosphaeria spartinae*, secondary metabolites

## Abstract

Due to a rate increase in the resistance of microbial pathogens to currently used antibiotics, there is a need in society for the discovery of novel antimicrobials. Historically, fungi are a proven source for antimicrobial compounds. The main goals of this study were to investigate the fungal diversity associated with sea foam collected around the coast of Prince Edward Island and the utility of this resource for the production of antimicrobial natural products. Obtained isolates were identified using ITS and nLSU rDNA sequences, fermented on four media, extracted and fractions enriched in secondary metabolites were screened for antimicrobial activity. The majority of the isolates obtained were ascomycetes, consisting of four recognized marine taxa along with other ubiquitous genera and many ‘unknown’ isolates that could not be identified to the species level using rDNA gene sequences. Secondary metabolite isolation efforts lead to the purification of the metabolites epolones A and B, pycnidione and coniothyrione from a strain of *Neosetophoma samarorum*; brefeldin A, leptosin J and the metabolite TMC-264 from an unknown fungus (probably representative of an *Edenia* sp.); and 1-hydroxy-6-methyl-8-hydroxymethylxanthone, chrysophanol and chrysophanol bianthrone from a *Phaeospheria spartinae* isolate. The biological activity of each of these metabolites was assessed against a panel of microbial pathogens as well as several cell lines.

## Introduction

Historically, sea foam has been investigated by marine mycologists as an inoculum source, particularly for obtaining the highly distinctive appendaged conidia of marine arenicolous ascomycetes (Kohlmeyer and Kohlmeyer 1979; Koehn 1982; Kirk 1983; Hyde and Jones 1989). Sea foam forms by the agitation of seawater containing dissolved organic matter (such as proteins, lignins and lipids) where the churning action of breaking waves in the surf zone traps air, forming persistent bubbles which adhere together creating a foam. Periods of strong onshore winds augmented by humid, overcast conditions facilitate the production of sea foam (Koehn 1982). During sea foam formation, a large number of fungal propagules become occluded within the air bubbles formed as the waves break upon the shoreline and the bubbles percolate through the shoreline sand (Hyde and Jones 1989). From previous surveys of fungal diversity observed from sea foam, the most common obligate marine species isolated from subtropical regions were *Corollospora* spp. (including the *Varicosporina ramulosa* anamorph) and *Carbosphaerella* spp. (Koehn 1982); whereas the more common species isolated from temperate waters also included *Corollospora* spp. (and *V. ramulosa*) along with *Asteromyces cruciatus, Dendryphiella arenaria*, and several *Phaeosphaeria* spp. (Kirk 1983). It is important to note that during these surveys the fungal propagules obtained from sea foam were not limited to marine fungi in particular; rather, conidia of terrestrial species that are often blown in from the shoreline and ubiquitous fungi naturally growing in the intertidal zone or blown in, are also frequently isolated (Koehn 1982; Kirk 1983).

The objective of this study was to survey the fungal diversity obtained from sea foam collected along the shoreline of Prince Edward Island (PEI) and to screen unique fungal isolates of interest for the production of secondary metabolites with a broad range of industrial applications. This manuscript focuses upon the antimicrobial activities observed from screening fungal extracts against a panel of six microbes including methicillin-resistant *Staphylococcus aureus* (MRSA) and vancomycin-resistant *Enterococcus faecium* (VRE); drug-resistant bacteria that are present and problematic in hospitals due to their resistance to first line antibiotic treatments and therefore are of particular interest to the pharmaceutical industry. Representative isolates of both marine lineages and potential novel taxa (based on analysis of ITS and nLSU rDNA gene sequence queries) obtained from sea foam were fermented under four media conditions, and culture extracts were generated using organic solvents. Culture extracts were fractionated using solid-phase extraction columns prior to screening for biological activity. Presented here is the diversity of fungi isolated from sea foam and an examination of several antimicrobial natural products produced from this resource.

## Materials and methods

### Collection and isolation

Sea foam resting on the water’s surface at the shoreline from five different locations around coastal PEI was collected into 50 mL sterile conical tubes in May and August 2011. Once the foam had settled in the tubes, an equal amount of sterile water containing 0.2 g/L chloramphenicol and 18 g/L Instant Ocean was added and a dilution series (100-, 1000- and 10,000-fold dilutions) was then prepared using sterile seawater (18 g/L Instant Ocean) containing 0.1 g/L chloramphenicol. A 10 µL aliquot of each dilution was pipetted into each well of 48-well plates containing YM-IO agar (1 mL of agar/well) and plates were incubated at 22°C and monitored daily over a 1-month period (for all media compositions in this manuscript refer to Supplementary Materials and Methods). Emerging fungal colonies were aseptically transferred onto Petri dishes containing YM-IO agar. After obtaining a pure isolate, seed inoculum was prepared by excising cubes (1–3 mm^3^) from an actively growing culture into 15 mL of yeast extract-maltose medium in a 50 mL test tube and incubated at 22°C, 200 rpm for 5 d, after which 500 µL of mycelial suspension was removed for DNA extraction and the remainder reserved to inoculate fermentations.

### DNA extraction and PCR amplification

Genomic DNA was obtained from all strains using a Fungi/Yeast Genomic DNA isolation kit (Norgen Biotek) according to the manufacturer’s protocols. Double-stranded copies of the ITS and nLSU rRNA gene were obtained by PCR amplifications (for PCR conditions see Supplementary Materials and Methods). Primers used for the ITS rDNA gene were ITS-1 and ITS-4 (White et al. 1990) and for the nLSU rDNA gene were LROR and LR7 (Rehner and Samuels 1994, Vilgalys and Hester 1990). PCR amplicons were checked for correct length and concentration by electrophoresis.

### DNA sequencing and sequence alignment

**Figure 1.**
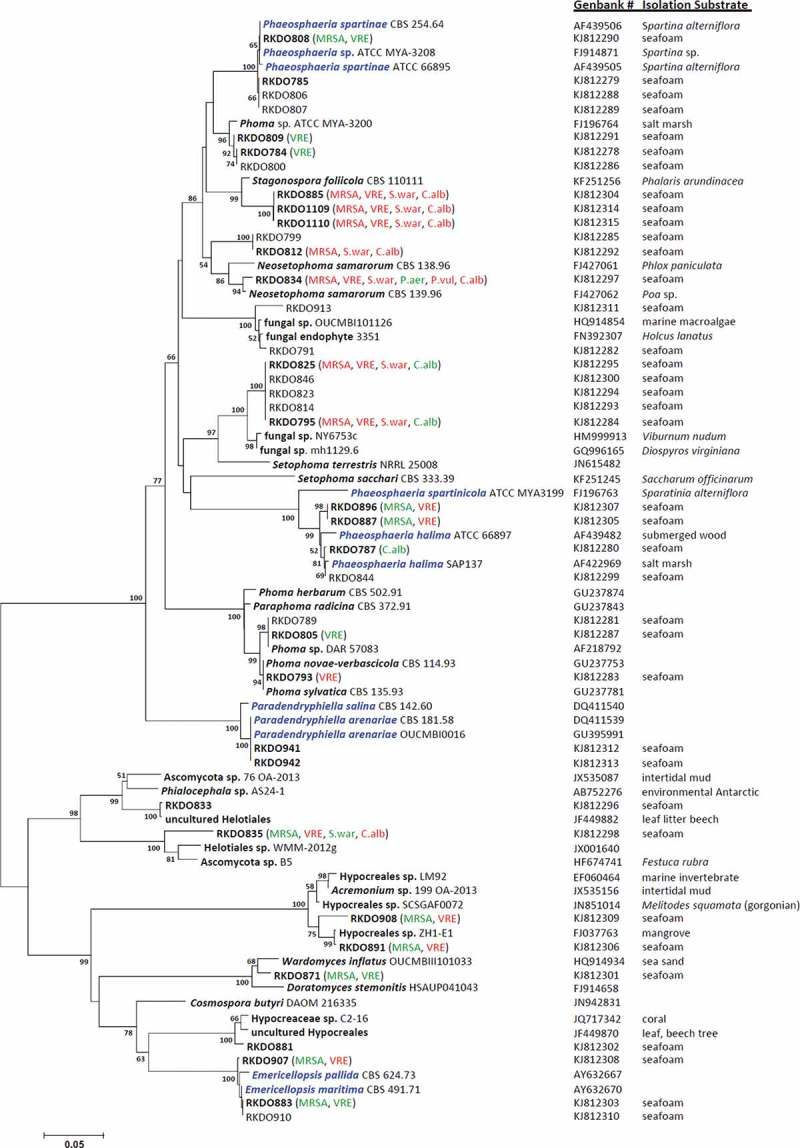
Bootstrap consensus tree inferred from 2000 replicates generated using the neighbor-joining method. Evolutionary distances were computed using the Maximum Composite Likelihood method. Numbers denote the percentage of replicate trees in which the associated taxa clustered together in the bootstrap test; only confidence values above 50% are indicated. RKDO isolates in bold font represent isolates that were screened for antimicrobial activity. Observed antimicrobial activities tested at 250 μg/mL are presented in brackets behind the isolate number (green = > 60% inhibition, red = > 80% inhibition, MRSA = methicillin-resistant *Staphylococcus aureus*, S.war = *S. warneri*, VRE = vancomycin-resistant *Enterococcus faecium*, P. aer = *Pseudomonas aeruginosa*, P. vul =* Proteus vulgaris*, and C.alb =* Candida albicans*). Species names in blue font represent true marine lineages.

The ITS and nLSU amplicons were sequenced at Eurofins MWG Biotech on a 3730xl DNA Analyzer coupled with BigDye Terminator v. 3.1 Cycle Sequencing reagents, Applied Biosystems (ABI). Sequences were compared with other fungal DNA sequences from the National Center for Biotechnology Information’s GenBank sequence database using a Blastn search algorithm (nLSU sequences were trimmed to include only the D1/D2 region to improve Blastn search results). Results from the Blastn search were carefully reviewed and species identities were chosen for isolates based on sequence homology congruence with accessioned fungal strains. Isolates lacking species level congruence were identified to the lowest taxonomic ranking that demonstrated congruence (genus, order, etc.). Isolates representing species of accepted marine lineages and those for which identification remained at the generic level or greater were selected for phylogenetic analysis, while isolates representing ubiquitous and accepted terrestrial fungal taxa were omitted. Sequence files of the closest match obtained from the Blastn search in GenBank for each isolate were included in separate phylogenetic analyses of the ITS and nLSU rDNA genes using the software Molecular Evolutionary Genetics Analysis v. 5 (MEGA5; Tamura et al. 2011). Sequences were aligned using the ClustalW algorithm, with a DNA Gap Open Penalty = 15.0, DNA Gap Extension Penalty = 6.66 and a delay divergent cutoff of 30%. Alignments were refined by manual correction when needed. Cluster analysis was carried out using the neighbor-joining method employing the maximum composite likelihood model using pairwise deletion and the clade stability was evaluated using the bootstrap method (*n* = 2000 bootstrap replications). Examined sequences were accessioned in GenBank (see Figure 1 and Supplementary Figure 1 for accession numbers).

### Fermentation and extraction

Three media were selected for liquid fermentations: CYA-IO, MMK2-IO, and YES-IO (all media compositions are included in Supplementary Materials and Methods). For 15 mL liquid fermentations, each 25 mL glass tube was inoculated with 500 μL of seed inoculum, capped and incubated under stationary conditions at 22°C for 14 d at an incline of 20 degrees above horizontal. Solid fermentations were carried out on a rice-based medium in 250 mL Erlenmeyer flasks, inoculated with 1.5 mL of seed inoculum and incubated under stationary conditions at 22°C for 21 d.

After incubation, fermentations were visually inspected for purity prior to extraction. For liquid fermentations, the mycelial mat and the broth in each tube were separated. The mycelial mat was cut into small pieces to increase efficiency of the extraction. Amberlite™ XAD Polymeric resin and Diaion^®^ HP-20 resin (activated with MeOH as per manufacturer’s instructions, Sigma Aldrich) were added (8 mL) to each broth prior to shaking at 200 rpm for 1 h. The resins were then removed, washed twice with distilled water and vacuum-filtered through Whatman^®^ #3 filter paper. The resin and mycelia were extracted with 40 mL of a 1:1 (v:v) ethyl acetate:methanol, shaken (200 rpm) for 1 h. Fungal colonies on solid fermentations were disrupted using a sterile spatula and extracted with 40 mL of EtOAc:MeOH (1:1) shaken at 150 rpm for 1 h. Organic extracts from both liquid and solid fermentations were individually vacuum-filtered through Whatman #3 filter paper and dried using a GeneVac vacuum evaporating system (model: EZ-2 MK2) prior to fractionation.

The resulting extracts were fractionated on Thermo HyperSep C_18_ Sep Pack columns (500 mg C_18_, 6 mL column volume) using a vacuum manifold by eluting with 14 mL of each of the following solvent systems: 20% MeOHaq (fraction 1), 50% MeOHaq (fraction 2), EtOH (fraction 3), and 50% MeOH/50% DCM (fraction 4). The eluent representing fractions 2–4 were retained and dried down using a GeneVac (model: EZ-2 MK2) evaporating system, weighed and submitted for antimicrobial testing, and analyzed by high performance liquid chromatography-high resolution mass spectrometry (HPLC-HRMS).

### General experimental procedures

Optical rotations were measured on a Rudolph Autopol III polarimeter using a 5 cm microcell (1 mL). NMR spectra were obtained on a 600 MHz Bruker Avance III NMR spectrometer. HPLC-HRMS data using a Kinetex 1.7 µm C_18_ column (Phenomenex, 50 × 2.1 mm) were recorded on Accela Thermo equipment with hyphenated HRMS-ELSD-UV detection: Exactive mass spectrometer (Thermo) fitted with an ESI source, a photodiode array detector, and a low-temperature evaporative light-scattering detector (LT-ELSD Sedex 80) Sedex 80. Automated flash chromatography was performed on a Teledyne Combiflash Rf200 using C_18_ or Diol RediSep columns. HPLC purifications were carried out on a Thermo Surveyor coupled with an ELSD Sedex 5 and a Waters auto-purification system with a UV detector, an ELSD and a sample manager–fraction collector.

### Scale-up fermentations of RKDO795, RKDO785, and RKDO834

Strains selected for scale-up fermentation were grown in 9 cm Petri dishes containing one of three media types (potato dextrose agar (PDA), Czapek yeast extract agar (CYA), and yeast extract sucrose agar (YES)) both containing and excluding sea salts (18 g Instant Ocean^®^ Sea Salt). Plates were incubated at 22°C and monitored over a period of 6 wks for sporulation to confirm the identifications made from sequence congruency with observed conidia ontogeny. In the case of RKDO834 several strains of *Neosetophoma samarorum* obtained from the Centraalbureau voor Schimmelcultures (CBS139.96, CBS568.94, and the type strain CBS138.96) were also cultured under the same growth conditions to allow for a phenotypic comparison. These strains were also fermented, extracted, and fractionated under the same conditions as described above and fractions were profiled by HPLC-HRMS.

Prior to large-scale fermentations, biological activity-guided fractionation was carried out on fraction 3, the active fraction obtained from the rice fermentation of RKDO834 generated during the initial antimicrobial screening. Fraction 3 was first fractionated using solid-phase extraction and then by preparative reverse-phase HPLC to yield pure epolone B (**A1**, 0.44 mg), pycnidione (**A2**, 3.47 mg), and epolone A (**A3**, 0.64 mg). Scaled-up fermentations of strain RKDO834 were carried out twice, first using 40 tubes of MMK2 medium and then in 10 flasks of Rice medium with the same volumes and growth conditions used in the preliminary screening. The mycelial mats from the MMK2 fermentations were removed from the fermentation broth and both were separately extracted with EtOAc. The resulting EtOAc MMK2 broth extract was further fractionated by reversed-phase HPLC to yield pure coniothyrione (**A4**, 2.3 mg). The rice fermentation was extracted using EtOAc fractionated by automated flash chromatography (Combiflash) yielding an enriched fraction of dehydroxy-pycnidione (**A5**, 32 mg). Each pure compound was evaluated for both antimicrobial and cytotoxic activities. Chromatography conditions used for compound isolations are provided in detail in Supplementary Materials and Methods.

The scaled-up fermentation of strain RKDO795 was carried out in 10 flasks of rice medium following the same growth conditions and extraction protocol as described previously. The dried EtOAc–MeOH extract from strain RKDO795 was then partitioned using successive liquid:liquid extractions and tested for biological activity. Biologically active compounds were isolated by fractionation using flash chromatography followed by normal-phase HPLC to afford leptosin J (**B1**, 5.3 mg) and reversed-phase HPLC yielding brefeldin A (**B2**, 9.1 mg) and metabolite TMC-264 (**B3**, 7.4 mg). Similarly, the scaled-up fermentation of strain RKDO785 was carried out in 10 flasks of rice medium following the same growth conditions and extraction protocol as described previously. The dried EtOAc–MeOH extract from RKDO785 was partitioned using successive liquid:liquid extractions and subsequently fractionated by flash chromatography followed by reversed-phase HPLC. Bioassay-guided fractionation yielded the following compounds: 1-hydroxy-6-methyl-8-hydroxymethylxanthone (**C1**, 0.66 mg), chrysophanol (**C2**, 3.17 mg), chrysophanol bianthrone (**C9**, 3.18 mg), and two uncharacterized bianthrone analogues **C7** (0.20 mg) and **C8** (0.25 mg). Antimicrobial and cytotoxic activities were evaluated for each compound. Liquid:liquid extraction and chromatography conditions used for compound isolations are provided in detail in Supplementary Materials and Methods.

### Antimicrobial assay

All microbroth antibiotic susceptibility testing was carried out in triplicate in 96-well plates in accordance with Clinical Laboratory Standards Institute testing standards (NCCLS, 2003) using the following pathogens: MRSA ATCC 33591 (MRSA), *S. warneri* ATCC 17917, VRE EF379 (VRE), *Pseudomonas aeruginosa* ATCC 14210, *Proteus vulgaris* ATCC 12454, and *Candida albicans* ATCC 14035. Extract fractions were assayed at 250 µg/mL while pure compounds were serially diluted to generate a range of 12 concentrations (128–0.0625 µg/mL) in a final well volume concentration of 2% DMSO(aq). Each plate contained eight uninoculated positive controls, eight untreated negative controls, and one column containing a concentration range of a control antibiotic (vancomycin for MRSA, and *S. warneri*, rifampicin for VRE, gentamycin for *P. aeruginosa*, ciprofloxacin for *P. vulgaris*, or nystatin for *C. albicans*). The optical density of the plate was recorded using a Thermo Scientific Varioskan Flash plate reader at 600 nm at time zero and then again after incubation of the plates for 22 h at 37°C. After subtracting the time zero OD600 from the final reading, the percentages of microorganism survival relative to vehicle control wells were calculated.

### Cytotoxicity assay

Human foreskin BJ fibroblast cells (ATCC CRL-2522), adult human epidermal keratinocytes (HEKa), and human breast adenocarcinoma cells (ATCC HTB-26) were grown to 80% confluency (growth conditions are provided in Supplementary Materials and Methods), the cells were counted, diluted, and plated into treated 96-well cell culture plates. The BJ fibroblast and HEKa cells were plated at a cell density of 10,000 cells per well and the HTB-26 cells were plated at cell density of 5000 cells per well in 90 µL of respective growth medium (without the addition of antibiotics) and incubated for 24 h to allow cells to adhere to the plates before treatment. Pure compounds were tested in triplicate in serial dilutions of eight concentrations ranging from 128 µg/mL to 1 µg/mL per well (final well volume of 100 µL, 1% DMSO per well). Each of the cell lines were incubated at 37°C in a humidified atmosphere of 5% CO_2_; the BJ fibroblast and HEKa cells for 24 h and the HTB-26 cells for 72 h. Each plate contained four uninoculated positive controls, four untreated negative controls, and one column containing a concentration range of zinc pyrithione or doxorubicin. Alamar blue was added, 24 h after the treatment, and fluorescence was monitored using a Thermo Scientific Varioskan Flash plate reader at 560/12 excitation, 590 nm emission both at time zero and 4 h after Alamar blue addition. The inferred percentage of cell viability relative to vehicle control wells were calculated after subtracting the time zero emission 590 nm measurement from the final reading and the IC_50_ was determined.

## Results

### Fungal diversity and preliminary screening

Blastn search results of ITS and nLSU rDNA gene sequences allowed for the identification of most of the isolates obtained from the sea foam. The majority of the isolates obtained were ascomycetes (representing 44 species) while only two species were identified as basidiomycetes and two as zygomycetes. From the total number of species isolated in this survey (*n* = 48 species), the following were either considered as ubiquitous or historically associated with terrestrial substrata, such as wood, plants, insects, and soil: *Alternaria alternata, Aspergillus fumigatus, Beauveria brongniartii, Bipolaris sorokiniana, Botryotinia fuckeliana, Cadophora luteo-olivacea, Cadophora malorum, Chalara piceae-abietis, Cistella acuum, Cladosporium* spp., *Drechslera dematoioidea, Epicoccum nigrum, Fibulorhizoctonia* sp., *Leptospaerulina australis, Mucor hiemalis, Myrothecium gramineum, N. samarorum, Penicillium* spp., *Peniophora aurantiaca, Pilidium concavum, Tolypocladium inflatum, Trichoderma harzianum*, and *Umbelopsis isabellina*. The remaining isolates, represented either species associated with lineages of marine fungi (*Paradendryphiella arenaria, Emericellopsis maritima, Phaeosphaeria halima*, and *Phaeospheria spartinae*) or were classified as unknown as they could not be identified to the species level using their ITS and nLSU sequences. All isolates representing species in these two categories were of considerable interest for the purpose of this study; therefore approximation of their taxonomic affiliations were visualized in a phylogenetic analysis of their ITS sequences and those of their associated closest matches obtained from GenBank (see Figure 1; nLSU phylogeny is provided in the Supplementary Materials and Methods).

All of the marine isolates, with the exception of the *P. arenaria* isolates RKDO883 and RKDO910, produced secondary metabolites that had an antimicrobial effect against one or more of the pathogens (see Figure 1). Extracts from isolates RKDO883 and RKDO910, representative of the marine species *E. maritima* (with a 99% ITS and 100% nLSU sequence homology), were active against both MRSA and VRE (inhibiting MRSA and VRE by 60% at a concentration of 250 µg/mL). Both isolates RKDO808 and RKDO785, which were representative of the marine species *P. spartinae*, produced different antimicrobial activity profiles. Fermentation extracts from isolate RKDO808 cultured on the rice medium demonstrated antimicrobial activity (70% growth inhibition against MRSA at 250 µg/mL and 70% inhibition against VRE at 50 µg/mL), whereas all of the culture extracts of isolate RKDO785 tested were inactive in the antimicrobial screening. Several other isolates (RKDO787, RKDO844, RKDO887, and RKDO896) were identified as *Phaeosphaeria halima*, based on ITS sequence homology with several different isolates of *P. halima*. Enriched culture fractions from representative isolates were found to have activity against both MRSA (60% inhibition at 250 µg/mL) and VRE (90% inhibition at 50 µg/mL). Similar to the representative isolates screened from marine lineages, the majority of the isolates classified as unknown lineages also produced fermentation extracts exhibiting an antimicrobial effect (see Figure 1). From this survey, three unique clades (A–C) from the ITS sequence phylogeny were selected for the follow-up chemical analysis.

### Isolation of secondary metabolites from RKDO834 (*Neosetophoma samarorum*)

Clade A, represented by isolate RKDO834, produced culture extracts that demonstrated a broad range of antimicrobial activity against both of the Gram positive, drug-resistant bacteria MRSA and VRE, the Gram negative bacterium *Proteus vulgaris*, and the yeast *Candida albicans*. A Blastn search of the ITS rDNA gene from RKDO834 in GenBank resulted in a tentative taxonomic identification of the strain as *N. samarorum* based upon a 99% similarity match (492/495 bases with no gaps) with that of *N. samarorum* strain CBS139.96. Comparison of the ITS rDNA to that of the type strain of species, CBS138.96, yielded a similarity match of 98% (484/495 bases with no gaps). A morphological comparison of strain RKDO834 compared to that of the type strain as well as additional strains (CBS139.96, CBS568.94) confirmed the identification of RKDO834 as *N. samarorum*. Moreover, secondary metabolite production by RKDO834 and the CBS strains was observed to be consistent by comparison of LC-HRMS profiles. Although the marine origin of our *N. samarorum* strain is dubious (as previous taxon isolation has been limited to terrestrial substrata), we were interested in the secondary metabolite production of RKDO834 due to assay screening data; therefore fermentation extracts from this fungus were prioritized for further chemical investigations to determine and characterize the active constituents.

**Figure 2.**
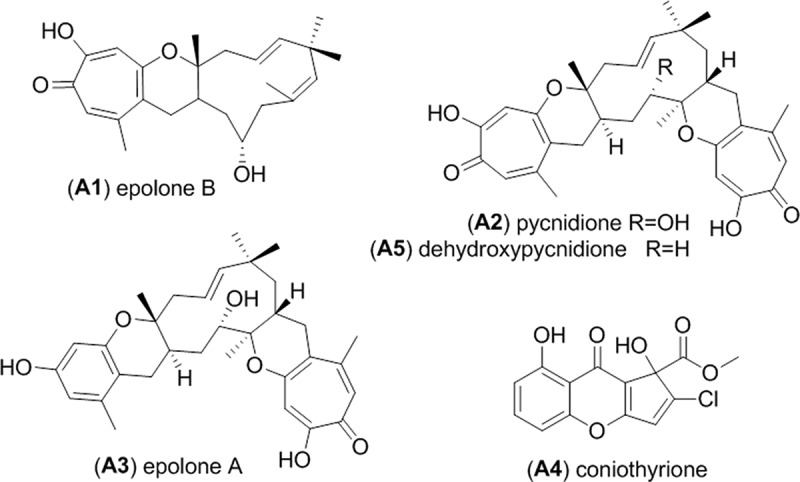
Metabolites isolated from *Neosetophoma samarorum* strain RKDO834.

Antimicrobial activities from the preliminary screening performed on the HyperSep C_18_ fractions were observed for fractions 3 and 4 of the rice medium fermentation extract from the strain RKDO834. Fraction 3 was selected for further bioassay-guided fractionation and the final HPLC purifications afforded three metabolites epolone B (**A1**), pycnidione (**A2**), and epolone A (**A3**) (Figure 2), all of which exhibited antimicrobial activity. Compound **A1** was determined by HRMS analysis to have the pseudomolecular ion [M + H]^+^ of *m/z* 385.2375 (calculated for C_24_H_33_O_4_^+^, 385.2373). NMR and optical rotation data were consistent with literature data confirming the metabolite as epolone B (Cai et al. 1998). Compound **A2** displayed an observed HRMS [M + H]^+^ ion *m/z* 549.2853 (calculated for C_33_H_41_O_7_^+^, 549.2847) and the NMR and optical rotation data were in agreement with those reported in the literature, confirming the identity of the compound as pycnidione (Harris et al. 1993). Compound **A3** was determined by HRMS analysis to have the pseudomolecular ion [M + H]^+^ *m/z* 521.2898 (calculated for C_32_H_41_O_6_^+^, 521.2897). NMR and optical rotation data matched with literature values for epolone A (Cai et al. 1998).

The scaled-up fermentation of RKDO834 in both MMK2 and the rice medium led to the isolation and the characterization of two additional metabolites (Figure 2). From the fermentation MMK2 broth, two consecutive HPLC purifications afforded coniothyrione (**A4**). The HPLC-HRMS analysis demonstrated an [M + H]^+^ ion at *m*/*z* 309.0161 (calculated for C_14_H_10_ClO_6_^+^, 309.0160) and an [M + Na]^+^ ion at *m*/*z* 330.9979 (calculated for C_14_H_9_ClO_6_Na^+^, 330.9980). Analysis of the 1D and 2D NMR data confirmed the structure as coniothryione (Ondeyka et al. 2007). After an EtOAc extraction of the mycelia obtained from the rice fermentation, a Combiflash separation allowed the isolation of an enriched fraction of a dehydroxy analogue of pycnidione. HPLC-HRMS analysis demonstrated an [M + H]^+^ *m/z* 533.2902 (calculated for C_33_H_41_O_6_^+^, 533.2898) and the observed UV profile was in agreement with a reduced pycnidione analogue. The NMR comparison of **A5** with those reported for pycnidione clearly identify the absence of a hydroxyl group (*δ*_H_ 3.60 in CDCl_3_) in the C-11 position.

### Isolation of secondary metabolites from RKDO795

**Figure 3.**
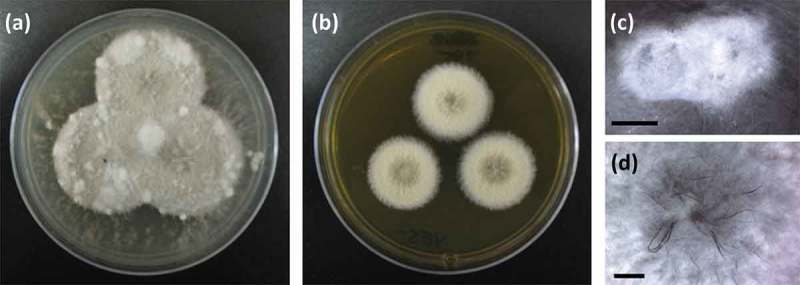
Phenotypic observations of strain RKDO795 (probable *Edenia* sp.) (a) colony morphology on CYA media (4 wks growth in 9 cm Petri dish) (b) colony morphology on YES media (4 wks growth in 9 cm Petri dish); (c) hyphal aggregate formed on PDA (scale bar = 2 mm); (d) darkened mycelial strands formed at the point of incoculation on YES (scale bar = 2 mm).

**Figure 4.**
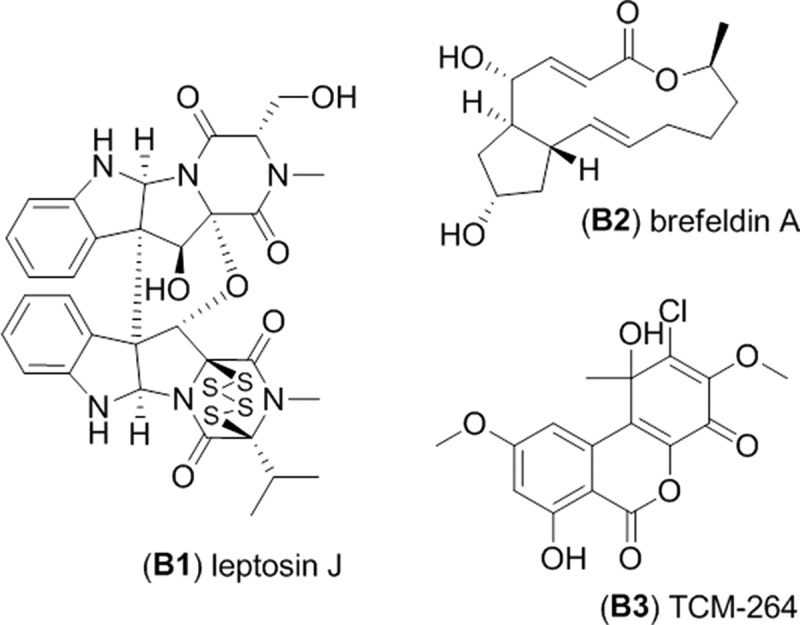
Metabolites isolated from strain RKDO795 (probable *Edenia* sp.).

Clade B, represented by the RKDO isolates 795, 814, 823, 825, and 846, was of interest based on a variety of activities that were observed as well as the taxonomic novelty suggested by ITS sequence comparison. All of the isolates produced woolly colonies of aerial mycelia that changed from white to dark gray with age on both PDA and CYA media (Figure 3a), producing white, loose mycelial strands darkening to dark brown/gray in the colony center and a black colony reverse. With age, cultures were found to produce white, hyphal aggregates, with a core of condensed, melanized hyphae (Figure 3c). Growth on YES media was more constrained and limited to smaller colonies with a velvety appearance (Figure 3b) that produced distinct, darkened, mycelial strands at the point of incoculation (Figure 3d). Growth rates were comparable in both the presence and absence of sea salts for all three media tested. Sporulation was absent on all of the colonies examined which made species identification based on conidia ontogeny impossible. Based upon ITS gene sequence homology, the closest identified fungal strain was the *Setophoma terrestris* strain NRRL25008 (JN615482.1) with a percent similarity of 89%. Blastn search using the D1/D2 region of the nLSU rDNA yielded a match of 99% similarity (595/601 base pair with 0 gaps) with the obscure fungus *Edenia gomezpompae* (strain CBS 124016; an ITS sequence for this strain is absent in GenBank). One of the strains, RKDO795 was selected for secondary metabolite characterization on rice medium as antimicrobial activity of enriched culture extract fractions demonstrated activity against both bacterial and fungal targets. Antimicrobial compounds were purified and characterized from the scaled up culturing of RKDO795 by bioassay-guided fractionation using flash chromatography and both reversed-phase and normal-phase HPLC (Figure 4). One of the compounds was identified as leptosin J (**B1**) by comparison of its measured HRMS, NMR, and optical rotation data with reported literature values (Takahashi et al. 1994a). Brefeldin A (**B2**) was also purified from the extract and identified by comparison of its HRMS, NMR, and optical rotation data with reported literature values (Glaser et al. 2000). A third compound TMC-264 (**B3**) was purified and identified on the basis of its HRMS, NMR, and optical rotation data and comparison with literature values (Sakurai et al. 2003a).

### Isolation of Secondary Metabolites from RKDO785 (Phaeosphaeria spartinae)

**Figure 5.**
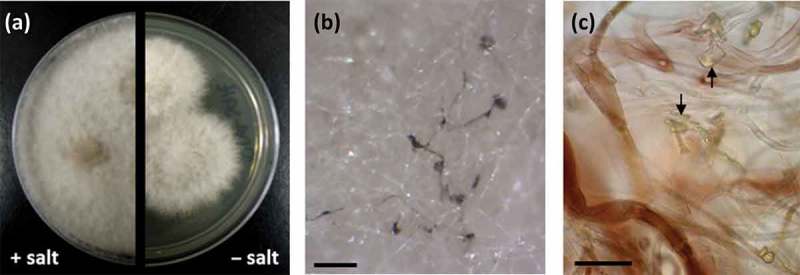
Phenotypic observations of strain RKDO785 (*Phaeosphaeria spartinae*) (a) colony morphology on PDA media, with and without sea salts (2 wks growth on a 9 cm Petri dish); (b) aerial mycelia demonstrating pigmentation (scale bar = 250 μm); (c) mycelia viewed in bright field microscopy at 100× magnification (scale bar = 20 μm); pigmentation is evident within mycelia (arrows indicate exudate crystals).

**Figure 6.**
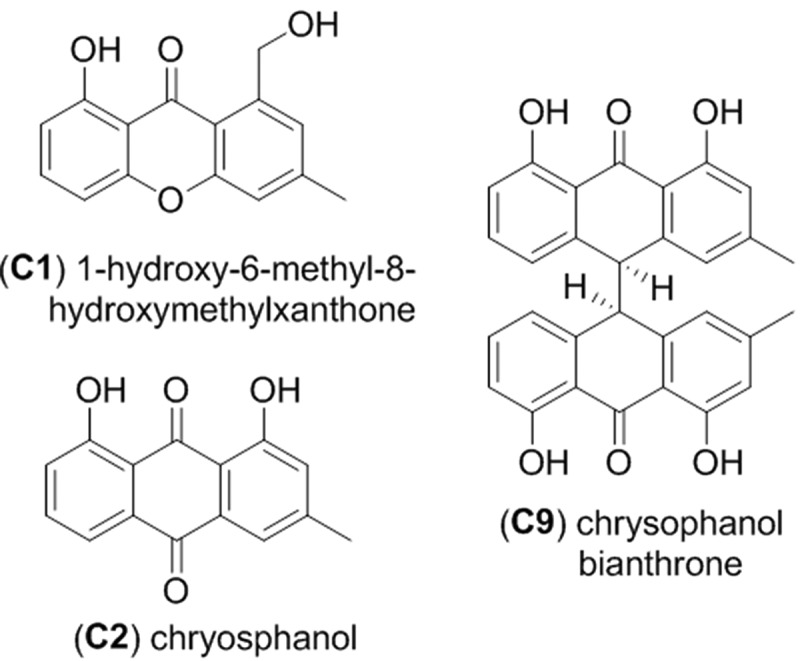
Metabolites isolated from *Phaeosphaeria spartinae* strain RKDO795.

Clade C, represented by a third group of isolates (RKDO785, RKDO806, RKDO807, and RKDO808), was identified by ITS sequence homology to be representative of the marine fungus *P. spartinae*. Isolate RKDO808 had an identical sequence homology with representative strains, while isolates RKDO785, RKDO806, and RKDO807 matched with 99% similarity (505/506 bases with 0 gaps for ATCC MYA-3208; 489/493 bases with 1 gap for ATCC 66895; and 473/474 bases with 0 gaps for CBS 254.64 respectively). All isolates produced colonies that were at least double in diameter length on media containing sea salts compared to the same media without sea salts (Figure 5a). Colonies were initially white and floccose, later becoming gray in color and had a dark reverse. With age, colonies often had, in parts, a red/brown to purple/brown tinge, due to the development of a pigmentation within the aerial mycelium (Figure 5b and c). Crystalized exudates were also observed in older cultures (Figure 5c). Hyphae were observed at times to be notably rough walled and hyphal coils were observed. Ascomata formation was absent from colonies cultured on all media tested (CYA, YES, and PDA). Although there was no antimicrobial activity observed for RKDO785 culture extracts, secondary metabolite production of isolate RKDO785 on rice medium was selected for further investigation due to an observed activity that was of direct interest to Nautilus Biosciences Canada Inc. (data not shown). The scaled-up fermentation of the fungal strain RKDO 785 allowed us to purify and identify three metabolites (**C1, C2**, and **C9**) by interpretation of 1D and 2D NMR experiments and comparison with literature data (Figure 6). Compound **C1** exhibited the pseudomolecular ion [M + H]^+^ *m/z* 257.0809 (calculated for C_15_H_12_O_4_^+^, 257.0808), and together with the observed NMR data, identified **C1** as the known metabolite 1-hydroxy-6-methyl-8-hydroxymethylxanthone (Ayer and Taylor 1976). In a similar manner, HRMS of compound **C2** supported a molecular formula C_15_H_10_O_4_ ([M–H]^–^ 253.0498, Δ −3.1ppm) and the ^1 ^H NMR data were in agreement with chrysophanol (**C2**) (Agarwal et al. 2000). Analysis of the NMR data of **C9** rapidly revealed the presence of two chrysophanol aromatic systems and the pseudomolecular ions [M–H]^–^ *m/z* 477.1331 (calculated for C_30_H_21_O_6_^–^, 477.1344) led to the proposed structure chrysophanol bianthrone (Alemayehu et al. 1993). LC-HRMS performed on other purified compounds confirmed the presence of two additional bianthrone analogues **C7** (C_30_H_20_O_8_) and **C8** (C_30_H_20_O_9_).

Fractions generated from RKDO808 grown on the rice medium were found to have an antimicrobial effect while fractions from RKDO785 did not. Differences in metabolite production between the two strains were evident when chromatograms were monitored in both positive and negative electrospray ionization. Four fatty acid compounds (**C3–C6**) were found to be exclusively produced by isolate RKDO 808. The origin of these fatty acids is unknown and it is possible that they are the result of a biological transformation of linoleic and oleic acid from the medium (as linoleic and oleic acids were observed both in the RKDO 808 extract as well as in the blank rice medium extract (rice medium control)). Analysis of HPLC-HRMS data recorded in positive and negative mode for these four metabolites supported the following molecular formulae C_18_H_30_O_3_ (**C3**), C_18_H_32_O_4_ (**C4**), C_18_H_32_O_3_ (**C5**), and C_18_H_34_O_4_ (**C6**). Antimicrobial activities that were observed from RKDO808 were associated with extract fractions enriched in these four fatty acids. A comparison between RKDO785 and RKDO808 in relation to the production of the compounds characterized in this study is provided in Table 1. Although the presence of the aforementioned fatty acids were limited to RKDO808, all of the characterized xanthone/anthraquinone/bianthrone metabolites were found to be produced by both strains (where production was confirmed by single ion monitoring mass spectrometry and by the characteristic UV–VIS absorption spectra for each of the metabolites in all of the fraction 3 fermentation extracts examined).

**Table 1.** Comparison of metabolites identified from *P. spartinae* strains RKDO785 and RKDO808 cultured in rice medium.

Compound	LCMS Rt (min)	[M + H]^+^, [M + Na]^+^, or [M–H]^–^	Metabolite class	RKDO 785	RKDO 808
**C1**	3.72	+257.0809 [M + H]^+^	Xanthone	+	+
**C2**	4.29	−253.0498 [M–H]^–^	Chrysophanol	+	+
**C3**	4.28	−293.2116 [M–H]^–^	Fatty acid	–	+
**C4**	4.28	+335.2192 [M + Na]^+^	Fatty acid	–	+
**C5**	4.52	−295.2271 [M–H]^–^	Fatty acid	–	+
**C6**	4.51	+337.2349 [M + Na]^+^	Fatty acid	–	+
**C7**	4.9	−507.1072 [M–H]^–^	Bianthrone	+	+
**C8**	5.15	−523.1025 [M–H]^–^	Bianthrone	+	+
**C9**	5.16	−477.1331 [M–H]^–^	Bianthrone	+	+

### Antibiotic and cytotoxicity screening

All of the metabolites that were purified and characterized during this survey were assayed for antimicrobial activity against our panel of Gram positive and Gram negative bacteria as well as the yeast *C. albicans* and cytotoxicity against a keratinocyte cell line (Tables 2 and 3). In some cases, the metabolites were also assayed for toxicity against a breast cancer cell line. Antimicrobial activity was observed for all of the compounds purified from RKDO834, with the exception of dehydroxy-pycnidione which was not run in the assay as the fraction containing the compound was not completely pure. Pycnidione and coniothyrione were observed to be more potent than epolones A and B against all of the pathogens tested with the exception of *P. aeruginosa*, for which none of the compounds demonstrated inhibitory activity. Pycnidione was the only compound that demonstrated a notable antifungal activity against *C. albicans* (MIC of 8 µg/mL and an IC_50_ value of 5 µg/mL), although epolone B did have an inhibitory effect. The compounds were also tested for cytotoxicity; all of which demonstrated varying degrees of activity against the cell lines tested (Table 2). The three metabolites purified from RKDO795 (leptosin J, brefeldin A, and TMC-264) varied in the antimicrobial effects observed. Both leptosin J and TMC-264 demonstrated antibiotic activity against Gram positive bacteria (leptosin J was particularly efficacious against MRSA with an MIC of 2 µg/mL and IC_50_ of 0.7 µg/mL) while brefeldin A was found to have an antifungal effect against *C. albicans*. All three compounds demonstrated cytotoxicity when tested against the keratinocyte, fibroblast, and the HTB-26 breast cancer cell lines (Table 2). Regarding the final three compounds purified in this study from RKDO785 (1-hydroxy-6-methyl-8-hydroxymethylxanthone, chrysophanol, and chrysophanol bianthrone), none of the metabolites had an antibiotic effect against our pathogen panel; however, 1-hydroxy-6-methyl-8-hydroxymethylxanthone was found to have a cytotoxic effect against all of the cell lines tested and chrysophanol bianthrone was found to have selective toxicity against the HTB-26 breast cancer cell line (MIC and IC_50_ value of 64 and 5.0 μg/ml, respectively).

**Table 2.** Biological activity of isolated metabolites against several bacterial pathogens along with antibiotics tested as a positive control. All assays were run in triplicate and averaged (expressed as µg/mL). If assay was not performed, table entry was left blank.

	MRSA		*S. warneri*		VRE		*P. vulgaris*		*P. aeruginosa*	
	MIC	IC_50_	MIC	IC_50_	MIC	IC_50_	MIC	IC_50_	MIC	IC_50_
Pycnidione	16	12	64	12	64	46	64	30	>128	>128
Epolone A	64	48	64	33	>64	44	>64	>64	>128	>128
Epolone B	64	48			>64	52	>64	>64	>128	>128
Coniothyrione	16	8	16	12	16	4	>64	30	>64	>64
Leptosin J	2	0.7	64	9	>128	128	>128	>128	>128	>128
Brefeldin A	>128	>128	>128	>128	>128	>128	>128	>128	>128	>128
TMC-264	64	14	64	23	>128	100	>128	>128	>128	>128
1-Hydroxy-6-methyl-8-hydroxymethylxanthone	>128	>128	>128	>128	>128	>128	>128	>128	>128	>128
Chrysophanol	>128	>128	>128	>128	>128	>128	>128	>128	>128	>128
Chrysophanol bianthrone	>128	>128	>128	>128	>128	128	>128	>128	>128	>128
Vancomycin	1.6	0.88	1	0.75						
Rifampicin					2	0.5				
Ciprofloxacin							0.25	0.063		
Gentamicin									2	1.25

**Table 3.** Biological activity of isolated metabolites along with positive controls (nystatin, zinc pyrithione, and doxorubicin) tested against the yeast *Candida albicans* and the cell lines HeKa (keratinocyte), BJ (fibroblast), and HTB-26 (breast cancer). All assays were run in triplicate and averaged (expressed as µg/mL). If assay was not performed, table entry was left blank.

	*C. albicans*		Keratinocyte		Fibroblast		HTB-26	
	MIC	IC_50_	MIC	IC_50_	MIC	IC_50_	MIC	IC_50_
Pycnidione	8	5	64	15	64	11		
Epolone A	>64	>64	32	13	32	14		
Epolone B	64	43	>128	50	>128	>128		
Coniothyrione	>64	>64	64	5				
Leptosin J	>128	>128	0.5	>0.5	16	0.5	0.5	>0.5
Brefeldin A	128	20	>64	0.5	>64	>64	>0.5	>0.5
TMC-264	>128	>128	4	1	16	13	8	2.8
1-Hydroxy-6-methyl-8-hydroxymethylxanthone	>128	>128	16	12	64	29	32	24
Chrysophanol	>128	>128	>64	>64	>64	>64	>64	>64
Chrysophanol bianthrone	>128	>128	>64	>64	>64	>64	64	4
Nystatin	1.6	0.92						
Zinc pyrithione			1	0.75	8	3		
Doxorubromycin							>16	2.5

## Discussion

Alignment of the ITS and nLSU rDNA nucleotide sequences amplified from RKDO834 were nearly identical to *N. samarorum* CBS139.96 for both of the genes examined and were similar to the type strain CBS138.96. Previous sequence alignment of the nSSU rDNA gene of three *N. samarorum* strains demonstrated the presence of an insertion within the nSSU gene for the type strain (CBS138.96) and CBS568.94 but absent in CBS139.96 (De Gruyter et al. 2010). The presence of this insertion within strains of *N. samarorum*, prompted de Gruyter and collaborators to suggest *Neosetophoma* represents a species complex. This insertion was also found to be absent in our strain RKDO834 (accessioned in Genbank as KJ812349). Regardless of observed phylogenetic differences between the strains, all of the *Neosetophoma* strains consistently produced the secondary metabolites: epolone A and B, pycnidione, dehydroxy-pycnidione and coniothyrione. Prior to our investigation, no chemistry had been reported from the species *N. samarorum*, or obligate synonyms of the name. The production of coniothyrione has only been reported once before from a strain of *Coniothyrium cerealis* in 2007 by Merck & Co. for observed Gram positive antibacterial activity that was attributed to the inhibition of protein and DNA synthesis (Ondeyka et al. 2007). As per literature reports, coniothyrione was found in this study to exhibit an antibiotic effect against the Gram positive bacteria MRSA, VRE, and *S. warneri* but did not inhibit the Gram negative bacterium *P. aeruginosa* and had a moderate bacterial static effect against *P. vulgaris*. Of the other metabolites isolated from RKDO834, epolones A and B and pycnidione were also tested for antimicrobial activity. Of the three, pycnidione was the most potent and had the widest spectrum of activity demonstrating antimicrobial effects against Gram positive bacteria, Gram negative bacteria, and fungi. The observed antibiotic activity can be associated to the tropolone moiety present in all three molecules. A tropolone is an aromatic seven-membered ring containing both keto and hydroxyl functional groups that has bacteriostatic and bactericidal activity for a wide range of bacterial species as well as antifungal and strong insecticidal activities (Trust 1975; Morita et al. 2003). Tropolones are currently found in the formulation of several oral care products (Pan et al. 2004a, 2004b). Differences in the observed potency of pycnidione compared to epolone A may be due to the presence of the di-tropolone moiety in pycnidione (Figure 2).

Both pycnidione and the epolones have a history of a broad range of biological activities against various drug targets and therefore have been the focus of several pharmaceutical natural product discovery investigations. Pycnidione was first discovered by Merck & Co. as a stromelysin inhibitor, with potential applications in the treatment of arthritis (Harris et al. 1993). Stromelysin is an enzyme postulated to cause cartilage degradation and one of the proposed causes for arthritis. Pycnidione inhibits the cleavage of β-casein by stromelysin with an IC_50_ of 31 µM (Harris et al. 1993). Since their discovery, antiplasmodial activity against *Plasmodium flaciparum* and anthelmintic activity against the parasitic worm *Hemonchus contortus* have been reported for both pycnidione and eupenifeldin (the stereoisomer of pycnidione) as well as anticancer activity against the cell lines KB and BC (Pittayakhajonwut et al. 2002; Ayers et al. 2008). The production of epolones A and B has only been reported once during investigations by OSI Pharmaceuticals for an alternative drug treatment to the use of recombinant human erythropoietin for anemia caused by chronic renal failure, cancer chemotherapy, and a variety of other disease states (Cai et al. 1998). Erythropoietin is the primary hormone responsible for regulating the proliferation and differentiation of immature erythroid cells and targets erythropoietin receptors on progenitor cells in bone marrow and other hematopoietic tissues (Cai et al. 1998). Epolones A and B and pycnidone induced erythropoietin gene expression five-fold at a concentration of 1–1.6 µM (Cai et al. 1998). No other biological activity has been previously reported for epolones A and B.

Reported production of pycnidione, epolones A and B is limited within the kingdom fungi to only a few species; however, these species belong to considerably different taxonomic orders: the Pleosporales, the Diaporthales, and the Sordariales (Harris et al. 1993; Pittayakhajonwut et al. 2002; Pornpakakul et al. 2007). Pycnidione was first discovered from an isolate that was originally characterized by morphology as an unidentified *Phoma* species (Harris et al. 1993) and subsequent sequencing of the D1/D2 region of the nLSU rDNA gene placed this isolate within the *Phaeosphaeriaceae* (Bills, personal communication 2012). Co-production of pycnidione and epolones A and B has only been reported once before (the only time for which epolones A and B have been reported) from an unidentified proprietary fungus OS-F69284 (belonging to the MYCOsearch collection, a subsidiary of OSI Pharmaceuticals) isolated from twigs obtained from a deciduous alluvial forest in Brazil (Cai et al. 1998). This isolate was characterized as producing floccose white sterile mycelia on malt extract agar, ¼ strength Czapek’s dox agar and yeast extract peptone agar. *N. samarorum*, although producing white sterile mycelia on malt extract agar, produces characteristic olivaceous green/buff yellow colonies when grown on yeast extract malt agar and oatmeal agar. Either a direct comparison of the culture morphology or representative rDNA gene sequences is required to prove/disprove if OS-F69284 is also a strain of *N. samarorum*.

From our culturing efforts, a definitive identification of isolate RKDO795 based on culture morphology was not possible. Blastn search results of the ITS rDNA gene were inconclusive, placing the isolate within the Pleosporales with proximity to the taxon *S. terrestris* that is currently classified within the *Phaeosphaeriacea* (Quaedvlieg et al. 2013). Sequence homology of the nLSU rDNA gene yielded a similar match with the obscure Pleosporalean fungus, *E. gomezpompae*. Currently only a taxon is described from the genus *Edenia*; from isolates obtained from senescent leaf spots (Crous et al. 2009). The cultural phenotype observed for isolate RKDO795, characterized in particular by the production of sterile colonies developing distinct hyphal strands within the colony center that turn brown with age, are shared with that of *E. gomezpompae* (Crous et al. 2009). In the original species description, the authors also noted the formation of sterile colonies of *E. gomezpompae* isolates on most media examined (Gonzalez et al. 2007). As sporulation was absent from RKDO795, a direct comparison of conidium ontogeny with that described for the type strain of *E. gomezpompae* could not be performed; therefore, our classification of RKDO795 at this time is that of a possible *Edenia* sp., at least until the type species is obtained and additional phylogenetically relevant genes are compared.

Isolate RKDO795 was found to produce the biologically active metabolite leptosin J which was first isolated from an unidentified *Leptosphaeria* strain that was isolated from seaweed (Takahashi et al. 1994a, 1994b). Due to observed polyphyly within the genus *Leptosphaeria*, many marine *Leptosphaeria* spp. have been reassigned to other genera based on phylogenetic studies and observed variations in culture micromorphology. For example, *L. orae-maris* is now synonymized with *Phaeosphaeria orae-maris* (Suetrong et al. 2009) and *L. obiones* is a synonym of *Byssothecium obiones* (Barr 2002). Discovery of leptosin J from RKDO795 is the only other report of leptosin J observed from a fungal isolate other than the original producing organism. As our producing strain (RKDO795) is classified within the *Phaeosphaeriaceae* (Quaedvlieg et al. 2013) it is possible that our producing strain is taxonomically similar to the original producing *Leptosphaeria* sp. strain. A direct comparison of our isolate with a comparative description of the producing strain or the strain itself, or a comparison of a taxonomically relevant gene sequence of the original producer is required to confirm this assumption. Unfortunately, the original report (Takahashi et al. 1994b) and all other subsequent reports of leptosin production by the *Leptosphaeria* sp. strain lack information regarding morphological criteria used for taxonomic identification; the producing strain was never accessioned into a public access culture collection and relevant ribosomal DNA sequences have not been reported.

Leptosins belong to a class of fungal toxins known as epipolythiodioxopiperazines; encompassing at least 14 different molecular groups, all of which contain a bridged polysulfide diketopiperzine ring. Examples of epipolythiodioxopiperazines such as gliotoxin, sirodesmin, and sporidesmin A are proven virulence factors associated with invasive mycosis of animals and plants (Gardiner et al. 2005). Epipolythiodioxopiperazines are also known to be fungitoxic; as observed in this study, leptosin J was found to have antifungal activity against the pathogen *C. albicans*. Leptosin J was also observed to be highly cytotoxic against keratinocyte and fibroblast cells and significant cytotoxicity of this molecule has been reported previously against P388 leukaemia cells (Takahashi et al. 1994a). The presence of the polysulfide bridge is responsible for the toxicity associated with epipolythiodioxopiperazine molecules (Müllbacher et al. 1986). The toxicity is thought to be mediated primarily by two mechanisms: molecular conjugation and subsequent inactivation of various cellular proteins via disulphide formation with susceptible protein thiol residues and by the generation of deleterious reactive oxygen species (such as superoxide or hydrogen peroxide) via redox cycling as the reduced molecule auto-oxidizes back to the disulphide form (Gardiner et al. 2005). Leptosins are currently being investigated as an anticancer therapeutic as the molecules inhibit DNA topoisomerase I and II and induce apoptosis by inactivation of Akt/protein kinase B (Yanagihara et al. 2005).

Brefeldin A and TMC-264 are two additional compounds isolated from RKDO795. Brefeldin A is a macrocyclic lactone produced by a variety of different ascomycetes (Bills et al. 2009). Various biological activities have been reported from brefeldin A (including antifungal and cytotoxicity as seen in this investigation), as the molecule inhibits organelle assembly in all eukaryotic cells. Brefeldin A interacts with the small G-protein Arf1 (ADP ribosylation factor1) and a guanidine exchange factor by binding reversibly at the interface of both proteins (Donaldson et al. 1992). The interaction induces morphological changes in several organelles, often leading to the disassembly of the Golgi complex and endoplasmic reticulum ultimately blocking vesicle-mediated protein transport between both organelles (Torii et al. 1995). As the effects of brefeldin A are reversible, this molecule has found application in biomedical research (Seehafer et al. 2013). The metabolite TMC-264, a chlorinated tricyclic heptaketide, was also isolated from RKDO795. TMC-264 was originally discovered for its potent, selective inhibitory affect against tyrosine phosphorylation of the transcription factor STAT6 (Sakurai et al. 2003b), a small molecule target for asthma pathogenesis (Hebenstreit et al. 2006). Signal transducers and activators of transcription (STATs) are a family of transcription factors that, upon tyrosine phosphorylation, transduce signals from the cellular membrane to the nucleus where they regulate gene expression upon binding to specific DNA sequences (Berg 2008). TMC-264 was first obtained from a *Phoma* sp. isolated from a soil sample collected in Japan (Sakurai et al. 2003b). The reporting authors provided a detailed morphological description of their fungus. Unfortunately, a direct morphological comparison with our isolate RKDO795 and their description was not possible as our isolate did not sporulate under a variety of culture conditions tested. Continued efforts are currently underway to try to induce sporulation of our strain to confirm its taxonomy.

Both *P. spartinae* isolates (RKDO785 and RKDO808) were found to produce xanthone, anthraquinone and bianthrone metabolites. All of these structurally similar molecules failed to produce notable antimicrobial activities; however, 1-hydroxy-6-methyl-8-hydroxymethylxanthone was found to be cytotoxic in each of the cell lines tested, while chrysophanol bianthrone was found to have selective toxicity against the HTB-26 breast cancer cell line. The xanthone metabolite 1-hydroxy-6-methyl-8-hydroxymethylxanthone was first isolated from the basidiomycete *Cyathus intermedius* (Ayer and Taylor 1976); however the production has been reported from ascomycetes from various orders including the *Eurotiales, Pleosporales, Pezizales* and the *Sordariales* (Hein et al. 1998; Höller et al. 2000; Pockrandt et al. 2012; Shen et al. 2012). The anthraquinone metabolite, chyrsophanol is one of the few polyketide natural products that occur in a broad range of eukaryotes as well as a few prokaryotes (Bringmann et al. 2009). Chrysophanol is reported to be associated with a broad range of biological activities, although this metabolite is most famous for its purgative effect (Srinivas et al. 2007). Treatment of J5 human liver cancer cells with chrysophanol has been reported to induce cell death (Lu et al. 2010), as chrysophanol is biotransformed in liver cells by a cytochrome P450-dependent oxidation into a more biologically reactive hydroxylated form (Mueller et al. 1998). In our cell line assay trials, selective toxicity was observed for the chrysophanol bianthrone isomer tested against the HTB-26 cell line while chrysophanol was inactive against all three cell lines tested at a concentration of 128 μg/mL. To the best of our knowledge, this is the first report of a chrysophanol bianthrone from a filamentous fungus.

All of the fungi isolated in this survey had the ability to grow in media containing sea salts. With the exception of *E. maritima*, all of the marine species isolated had previously been described from sea foam surveys from temperate waters carried out by Kirk (1983). One commonality shared between our survey and other similar sea foam fungal diversity studies (Koehn 1982; Kirk 1983) is the isolation of a substantial number of ubiquitous saprophytic and plant pathogenic genera. The presence of these ubiquitous and plant pathogenic fungi in the sea foam is a likely result of wind dispersal of spores produced on substrata present from neighboring fields, as sea foam is generated when strong onshore winds exist during times of overcast skies and humidity (Kirk 1983). Additional to the species identified in this survey (ubiquitous, plant pathogenic, and marine fungi), a considerable number of isolates obtained were classified as unknown based on ITS and nLSU sequence homology; some of which might represent marine fungi. Further examination of the micromorphology of the unknown species may give some insight as to their potential marine affiliation (i.e. presence of morphological adaptations to aid in the dispersal of spores in water, such as appendaged ascospores or ornamented conidia that are commonly associated with particular classes of marine taxa).

### Concluding remarks

Regarding the isolates obtained in this survey, sea foam was found to be an excellent source of inoculum which yielded a diverse range in fungi, many of which were unknown taxa that produced natural products exhibiting antimicrobial activity against relevant disease targets. The majority of marine fungi that were isolated were consistent with marine fungal taxa obtained in previous investigations with sea foam from temperate climates. Bioassay-guided fractionation of culture extracts from three prioritized isolates lead to the identification of 10 different biologically active secondary metabolites. With the exception of dehydroxypycnidione, all of these secondary metabolites had been reported previously; however, the producing organisms identified in this survey differed in taxonomy from the original reported producers. This study was the first to investigate and characterize secondary metabolite production from the taxon *N. samarorum*.
